# Economic Evaluation of Alcoholism Treatment

**Published:** 2006

**Authors:** Jeremy W. Bray, Gary A. Zarkin

**Affiliations:** Jeremy W. Bray, Ph.D., and Gary A. Zarkin, Ph.D., are health economists at RTI International, Research Triangle Park, North Carolina

**Keywords:** health services research, alcoholism treatment services research, health care costs, social and economic cost of AODU (alcohol and other drug use), treatment cost, AODU cost estimation problem, cost-effectiveness of AOD health services

## Abstract

Rising health care costs in recent years have increased pressures on providers, insurers, and policymakers to monitor the costs, cost-effectiveness, and cost–benefit of all health care services, including alcohol-related services. Without solid information regarding the economic implications of alcohol-related services, health insurance companies, managed care organizations, and policymakers may be reluctant to fund these services. As reviewed in this article, economic analyses—such as cost, cost-effectiveness, and cost–benefit analyses, including cost-offset studies—have been applied to alcoholism treatment outcomes research to provide such information. Methodological issues discussed here that concern these approaches will shape the future direction of economic analyses in the alcohol field.

Alcohol abuse and dependence as well as less severe alcohol-related problems are associated with significant social costs. For example, in 1998 the social costs of alcohol abuse and dependence were estimated to be $184.6 billion (Harwood 2000). Although alcohol-abusing drinkers and their families bear some of these costs (e.g., medical and legal costs), the non-abusing population also bears costs related to the adverse social consequences of problems such as alcohol-related motor vehicle crashes, crime, and increased health care costs. Because of these enormous societal costs, alcoholism^1^ is a major concern among policymakers and researchers.

Traditionally, policymakers and researchers in the alcohol field have focused much of their attention on the effectiveness of treatment. However, in recent years managed care organizations have placed increasing emphasis on cost containment, which requires that these treatments also be cost-effective, thereby necessitating greater use of economic analyses of these services. To investigate the role of such economic analyses, the National Institute on Alcohol Abuse and Alcoholism’s (NIAAA’s) National Advisory Council formed a Subcommittee on Health Services Research. In its report to the Advisory Council, the Subcommittee noted that studies on the cost and cost-effectiveness of alcoholism treatments are essential to ensuring that people with alcohol-related problems receive appropriate care and recommended that future research should compare the costs of alcoholism treatment programs with their outcomes and benefits (Subcommittee on Health Services Research 1997).

In light of the heightened need for and emphasis on understanding the economic value of alcoholism treatment services, researchers increasingly are conducting economic evaluations of these services. This article presents an overview of economic analysis methods that currently are applied to alcohol services research, such as cost analysis, cost-effectiveness analysis, and cost–benefit analysis, including cost-offset studies. For each type of analysis, an overview of standard methods is provided, and their advantages and disadvantages are briefly discussed. The article concludes with a discussion of the methodological issues shaping the future direction of economic analyses in alcohol research.

## Methods of Economic Analysis

In the alcohol field (as in other medical areas), the economic evaluation of health interventions involves three interrelated types of analyses, which are described further in the following sections:

- Cost analyses, which estimate the resources used to deliver alcoholism treatment services and the monetary value of those resources to various stakeholders (e.g., society, treatment providers, or government funding agencies).
- Cost-effectiveness analyses (CEAs), which are used to compare two or more treatment alternatives (one of which may be a no-treatment baseline) in terms of both costs and effectiveness.
- Cost–benefit analyses (CBAs), which directly compare the costs of a treatment with the monetary value of the resulting outcomes. Because CBAs measure both costs and outcomes in monetary terms, they facilitate comparisons of alcoholism treatment services with other types of investments in health and well-being.

For a summary of the features of these methods, see the table.

### Cost Analysis

A cost analysis is the first step in a full economic evaluation of a treatment intervention and provides critical information beyond its contribution to a CEA or CBA (Drummond et al. 1997). Ideally, cost analyses not only provide dollar estimates of the cost of alcoholism treatment services in a specific setting, but also offer information on the amount of resources (e.g., labor, facilities, and supplies) used in providing those services. Only with this additional information on resources can the results be applied to different circumstances and adjusted to account for rising or declining prices. Cost studies also identify the resources (e.g., labor) that account for the largest share of the overall cost of treatment. This information allows policymakers to identify critical cost components and facilitates sensitivity analyses that assess whether the results of a CEA or CBA are affected by changes in key assumptions. Finally, cost studies compare the relative cost of one service to another, allowing policymakers to better allocate scarce treatment resources.

To assess the relative economic merits of various alcohol services accurately, it is imperative that the costs of the interventions be measured from the same perspective using similar and appropriate methodologies. For example, accounting or actuarial cost estimation methods (which add up all expenses paid for by the program as well as all payments made to the program) focus on the actual expenses of a program. These approaches often can yield misleading conclusions about program costs, however, because they ignore the value of volunteered or other “free” resources, such as volunteer labor or donated space or equipment. As a result, a program may appear to be relatively inexpensive simply because its building space was donated at zero cost to the program and some labor was supplied by volunteers. In contrast, economic costing methodologies are based on opportunity costs—the value of a good or service in its next-best use. For example, the opportunity cost of volunteers’ time is the wage that those volunteers could earn if they worked in a paying job instead of volunteering their time. When the opportunity costs of volunteered goods and services are included in the cost analysis, decisionmakers get a more accurate view of all the resources necessary to replicate the program in question.

To ensure that program costs are consistently measured and all opportunity costs completely assessed, formal economic cost studies rely on systematic cost collection tools. The first such tool to be used in the alcohol and other drug (AOD) abuse field was the Drug Abuse Treatment Cost Analysis Program (DATCAP), which was developed in the early 1990s by economists at RTI International (French et al. 1997). This tool estimates the total and per-patient costs of an AOD abuse treatment program based on information on program revenue, patient flow, staffing, and other resource use. DATCAP has been used extensively in the AOD literature, and its systematic costing methodology facilitates comparison of program costs across a broad range of alcoholism treatment programs.

A limitation of DATCAP is that it estimates the per-patient cost of a treatment program without attempting to separate the costs of the individual component services (e.g., motivational interviewing, pharmacotherapy, or self-help programs) that comprise that treatment program. Service-level cost estimates are critical to sound policy decisions, however, because different treatment programs may use different combinations of services.

Recognizing DATCAP’s limitation, Zarkin and colleagues (2004) developed the Substance Abuse Services Cost Analysis Program (SASCAP), which includes two components:

- A cost survey (SASCAP–Cost) to collect data on program costs and revenue
- A labor allocation survey (SASCAP–Labor) to collect data on staff time allocated across various treatment services.

Using both components, researchers can estimate the costs of discrete treatment services as well as the average costs per patient. This new approach to estimating the costs of treatment services yields estimates that include not only the cost of direct labor but also the cost of indirect labor (e.g., administrative staff) and nonlabor resources. In addition, the indirect labor costs explicitly include the costs of administrative services (e.g., human resources, legal fees, billing, and marketing) provided to the program by a parent organization, which represents an advance over previous cost estimation studies.

Although tools such as DATCAP and SASCAP greatly facilitate economic cost studies of alcoholism treatment programs, standardized cost studies still pose many challenges for researchers. Perhaps the greatest of these is the burden on both researchers and program staff in terms of completing a standardized cost instrument. For example, DATCAP takes approximately 8 to 16 person-hours to complete (including both preparation and interview time) and should be administered by a trained economist (French 2003). SASCAP is less burdensome than DATCAP, but the time necessary to complete it still is a concern. Anecdotal evidence suggests that program staff spent an average of 4 to 5 hours completing both SASCAP components (Zarkin et al. 2004). However, SASCAP can be administered by noneconomists.

Although the term *cost study* has been used for more than a decade in the alcohol services research literature, formal economic cost analyses are a relatively new addition to alcohol services research. Most cost studies described in the alcoholism treatment literature are components of CEAs or CBAs. For example, formal cost studies have been discussed as part of economic evaluations of screening and brief intervention for hazardous alcohol use (e.g., Fleming et al. 2000; Wutzke et al. 2001). Increasingly, however, formal economic cost studies are being published as stand-alone analyses (e.g., Cisler et al. 1998; Zarkin et al. 2003, 2005).

### Cost-Effectiveness Analysis

CEAs compare two or more treatments using the cost-effectiveness ratio—the difference in costs between two interventions divided by the difference in outcomes (Gold et al. 1996). In other words, the cost-effectiveness ratio reflects how much more it costs to achieve one additional unit of outcome with one treatment intervention than with another intervention. For example, a cost-effectiveness ratio could show that alcohol intervention A costs $1,000 more than intervention B for every additional day of abstinence achieved.

Although the cost-effectiveness ratio is a useful summary of both the costs and effectiveness of an intervention, it does not necessarily provide decisionmakers with definitive policy recommendations. It is tempting to say that the intervention with the lowest cost-effectiveness ratio should be adopted, but if stakeholders value the outcome highly, then it may be more beneficial to use an intervention with a higher cost-effectiveness ratio. Economists refer to the implicit value that decisionmakers assign to an outcome as the decisionmakers’ willingness to pay for that outcome. The most desirable intervention among a set of mutually exclusive choices is the most effective intervention with an incremental cost-effectiveness ratio that is less than the decisionmaker’s willingness to pay for the intervention, excluding dominated interventions (Johannesson and Weinstein 1993; Bala and Zarkin 2002; Gold et al. 1996). An intervention may be dominated in either a simple sense (higher cost and lower effectiveness than another option) or in an extended sense (higher cost-effectiveness ratio than a more effective or less expensive option). For a clear and concise summary of the decision rules to use when interpreting CEAs, see Bala and Zarkin (2002).

The choice of outcome to be analyzed is a key issue when using CEAs. Some authors (e.g., Drummond et al. 1997) have argued that any clinical or policy-relevant outcome, such as percentage of abstinent days, can be used if it is relevant for all interventions under consideration. Sindelar and colleagues (2004), however, have posited that AOD treatments often yield numerous worthwhile outcomes which are not always correlated. As a result, the cost-effectiveness ratio for one outcome measure may yield a different policy implication than the ratio for another outcome measure. One solution to this problem is to use a common metric such as the quality-adjusted life year (QALY) as the outcome (Gold et al. 1996). QALYs assign a quality-of-life weight to each additional year of life generated by a treatment, with a weight of 1.0 indicating perfect health and a weight of 0 indicating death. An advantage of QALYs is that they can be used to compare a wide range of programs with each other, even services as diverse as alcoholism treatment and blood pressure screening. A problem with using QALYs, however, is that they can be difficult to estimate, especially for services which are expected to have only small effects on the patients’ quality of life in the near term.

CEAs have been employed in alcohol services research for a long time (for a historical review, see French 2001; for recent examples, see Chisholm et al. 2004; Corry et al. 2004; Kaskutas et al. 2004; Palmer et al. 2000). Most CEAs of alcohol services compare more intensive treatment (e.g., inpatient) with less intensive treatment (e.g., partial hospitalization) (e.g., McCrady et al. 1986; Kaskutas et al. 2004). More recent studies also have examined the cost-effectiveness of pharmacotherapies for alcohol dependence (e.g., Schädlich and Brecht 1998) or of screening and brief intervention (Wutzke et al. 2001). In general, most studies have found that less intensive treatments may be more cost-effective than more intensive treatments (French 2001). Some investigators have raised concerns about these conclusions, however, because the standard analytic methods used in these studies do not account for subgroups of patients who may benefit disproportionately from more intensive treatments (Bray et al. 2004).

### Cost–Benefit Analysis

Conceptually, CBAs not only calculate the costs of treatment but also place a dollar value on all outcomes (e.g., the value of avoided car crashes or reduced health care costs). As a result, the dollar value of the benefits associated with a treatment program can be directly compared with the dollar value of the program’s costs (Drummond et al. 1997). In practice, however, not all outcomes are assigned a dollar value (i.e., are monetized). Depending on which specific outcomes are monetized, CBA studies also may be referred to as cost-offset studies (which typically use only future health care costs as the benefits) and return-on-investment (ROI) studies (which typically monetize only benefits to the organization that funded the treatment). Because CBAs combine multiple outcomes into a single measure and allow direct comparison of costs to benefits, they often provide clearer guidance than CEAs on which treatment programs should be adopted—namely, those programs whose benefits exceed their costs. CEAs, in contrast, provide a ranking of competing alternatives but do not provide information on the extrinsic value of a treatment program independent of the alternatives.

**Table:** Methods for Analyzing the Economic Costs and Benefits of Health Services

Definition	Comments/Tools Used	Advantages	Disadvantages
**Cost Analysis**			
Is the first step when comparing costs of different services. Estimates the cost of delivering all alcoholism treatment services in a setting; estimates the resources needed to provide the treatment and the monetary value of the resources to society, government funding agencies, treatment providers, and other stakeholders.	Needs to include value of volunteered goods or labor to be informative. Relies on systematic data collection tools: Drug Abuse Treatment Cost Analysis Program (DATCAP)—the first such tool used; estimates total and per-patient costs of an alcohol and other drug abuse treatment. Substance Abuse Services Cost Analysis Program (SASCAP)—collects data on program costs and revenues as well as on direct and indirect labor costs.	Allows policymakers to identify critical cost components and facilitates sensitivity analyses that assess whether the results of a CEA or CBA are affected by changes in key assumptions.	Filling out the standardized cost instrument is burdensome for researchers and program staff.
**Cost-Effectiveness Analysis (CEA)**			
Compares two or more treatments using a cost-effectiveness ratio—the difference in costs between two interventions divided by the difference in outcomes. Is a measure of how much more it costs to achieve one unit of outcome with one treatment program than with another.	Usefulness depends on which outcome is analyzed because cost-effectiveness ratios for different treatment outcomes may have different policy implications. To address this issue, a common metric can be used: the quality-adjusted life year (QALY).	QALYs can compare a wide range of diverse programs. CEAs can yield a ranking of competing alternatives.	QALYs are difficult to measure, especially for services expected to have small effects. CEAs do not provide information on the value of a program independent of alternatives.
**Cost–Benefit Analysis (CBA)**			
Calculates the costs of treatment; also places a dollar value on all outcomes. Can show which programs have benefits that exceed their costs. Directly compares the costs of treatment with the monetary value of the outcomes. Combines all outcomes studied into one measure.	CBAs measure both costs and outcomes in monetary terms, which facilitates comparisons of alcoholism treatment services with other types of investments in health and well-being. CBAs usually put a dollar value on some outcomes, not all. Subtypes of CBAs include cost-offset studies (which only consider future health care costs as benefits) and return-on-investment studies (which consider only benefits to the organization funding the treatment).	CBAs can allow direct comparison of costs to benefits. May give clearer guidance than CEAs on which treatment to adopt.	Many outcome measures cannot be expressed in dollars. Some variables could be considered either benefits or costs. Benefits of some programs might be measured more readily than benefits of others, and some benefits may manifest only over several years after treatment. Results and implications depend on whether a narrow stakeholder (e.g., health care provider, health plan, employer) perspective or a broader societal perspective is used.

Various methods can be used to compare the benefits of a program with its costs. The two most commonly used methods are:

- Net benefit measures, in which the costs are subtracted from the benefits
- Benefit–cost ratios, in which the benefits are expressed as a percentage of the program costs.

The choice of CBA measure is largely driven by the context in which the analysis is conducted and by the audience for which the analysis is performed. Some authors caution against using a benefit–cost ratio approach, however, because deciding whether a given variable is considered a cost or a benefit can have a large impact on the final benefit–cost ratio (Drummond et al. 1997). For example, if a researcher considers car crashes a cost of alcohol use, then the dollar value of the car crashes suffered by a treatment program’s participants could be considered a cost of that program, and therefore would be included in the denominator of the benefit–cost ratio. Conversely, the researcher could consider avoided car crashes a benefit of the treatment program, in which case the dollar value of avoided car crashes would be included in the numerator of the benefit–cost ratio. Obviously, the final value of the benefit–cost ratio can differ greatly depending on whether the costs associated with car crashes are included in the numerator or the denominator. For this reason, it is suggested that analysts use the net benefit approach.

CBAs are not always easy to implement. For example, it may be challenging to estimate dollar values for all outcomes of the interventions because many clinically relevant outcomes cannot be expressed in dollars (e.g., reduced levels of alcohol use). Furthermore, it may not be possible to measure all relevant outcomes of an intervention, leading to an underestimate of the benefits of some interventions. If the benefits of one program can be monetized more readily than those of another program, a CBA of both programs may yield misleading results, and the wrong intervention may be adopted.

Because the choice of outcome measures can have such a great influence on the results of a CBA, researchers who want to conduct CBAs should examine the literature on the social costs of alcohol-related problems for guidance on which outcomes to measure and how to monetize them (e.g., Harwood et al. 1998). This approach ensures a minimal level of comparability across CBAs of varying alcohol services.

Another challenge with CBAs is that many of the benefits of alcohol services manifest over several years after treatment. Therefore, it is important that researchers who take into account future benefits (and costs) present all costs and benefits at their present value—an approach called discounting. Several texts provide guidance on the process of discounting and the choice of the discount rate to adjust future costs and benefits to their present value (e.g., Gold et al. 1996; Drummond et al. 1997).

Another key issue is the perspective from which the CBA is conducted. Historically, many CBAs of alcohol services have been conducted from the narrow perspective of one or more key stakeholders (i.e., employers, health plans, and State governments)—that is, the analysis included only costs and benefits to these stakeholders. For example, one form of CBA that has been widely used in the alcohol services research field is cost-offset analysis (Holder and Blose 1986, 1992; Holder and Hallan 1986; Goodman et al. 1991, 2000; Booth et al. 1997; Parthasarathy et al. 2001; Kane et al. 2004). Although the definition of “cost-offset” has varied among studies, the term suggests that the cost of alcoholism treatment may be offset by a reduction in future medical care use and health care costs (Holder and Blose 1992). Thus, these studies look at a narrowly defined net benefit of alcoholism treatment that considers only reductions in future health care utilization as the benefits of such treatment.

In contrast, Gold and colleagues (1996) strongly recommend that analysts adopt a broader societal perspective. In line with this recommendation, Fleming and colleagues (2000) applied the CBA approach to Project Trial for Early Alcohol Treatment (TrEAT), in which physicians administered brief interventions to problem drinkers in a randomized controlled trial. The authors performed the CBA both from a medical care system perspective and from a societal perspective. In this approach, the medical care perspective considered the decrease in future emergency department visits and hospitalizations as benefits, whereas the societal benefits perspective incorporated other benefits, such as reductions in alcohol-related traffic crashes, crimes, and legal proceedings. (See the article by Mundt in this issue for more on the economic analysis of Project TrEAT.)

Both narrow stakeholder perspectives and the broader societal perspective can yield useful results. For example, the societal perspective improves comparability across studies by providing a common perspective. Enumerating and monetizing *all* socially relevant outcomes, however, often is difficult. Furthermore, not all social outcomes are relevant to all stakeholders that sponsor alcohol services. Health care providers, workplaces, and schools all make decisions on funding alcohol services and have outcomes and costs that are unique to their organizations. Thus, both narrow stakeholder perspectives and a broader societal perspective are necessary to inform alcohol policy and service adoption.

## Conclusions

Because of the enormous social costs they impose, alcohol abuse and dependence as well as other alcohol-related problems are of major concern to policymakers and researchers, and considerable research into the effectiveness of alcoholism treatment has been performed as a result. Yet increasing health care costs demand that these treatments be cost-effective as well as efficacious. In light of the increased need for and emphasis on understanding the economic value of alcoholism treatment, economic analyses are playing a more prominent role in alcohol services research. Although previous research into the cost, cost-effectiveness, and cost–benefit of alcohol services has provided critically important and policy-relevant information, three emerging issues suggest that yet more economic evaluations of alcoholism treatment services are needed.

First, and perhaps foremost, as new treatment alternatives are developed, ongoing economic analyses must examine not only these new therapies but also existing therapies, both alone and in combination with the new therapies. For example, NIAAA has funded a CEA of the treatment approaches included in COMBINE, a multisite, randomized controlled trial that is assessing the efficacy of two pharmacotherapies (i.e., naltrexone and acamprosate) and psychotherapy, individually and in combination, in the treatment of alcohol dependence. COMBINE is one of the most ambitious clinical trials of alcoholism treatment ever undertaken, and its ancillary CEA should yield invaluable information on the costs and cost-effectiveness of combining behavioral therapies and pharmacotherapies for treating alcohol dependence. Many novel treatment approaches (e.g., pharmacotherapies, motivational interviewing) have promise both as stand-alone treatments and as additional services that can improve the effectiveness of existing treatment modalities. Therefore, ongoing economic evaluations of all available treatment options are necessary to ensure that scarce treatment resources are used most efficiently.

Second, recent improvements in economic costing methodologies combined with the relative lack of formal cost studies in the literature suggest that much more research is needed into the economic cost of existing alcoholism treatment services. Because many studies have used DATCAP to estimate the cost of AOD abuse treatment programs, further cost analyses of new alcoholism treatment methods using this approach may be needed so that the findings can be compared with findings in the existing literature. However, it is equally if not more important that cost studies be conducted using newer costing methods, such as SASCAP. Using these newer methods, cost studies can provide critical information on the cost of individual treatment services across modalities. This information will both improve allocation decisions and inform the next generation of CEA and CBA studies.

Finally, recent studies suggest that CEAs may be biased against less intensive forms of treatment if the effectiveness of treatment varies systematically with the severity of the patient’s condition (Bray et al. 2004). Many previous studies on the cost-effectiveness of alcohol services have compared the outcomes of patients receiving more intensive treatment services, such as inpatient care, with those of patients receiving less intensive treatment, such as partial hospitalization, and concluded that less intensive treatments are more cost-effective. If the effect of treatment depends on patient severity, however, it is possible that the outcomes obtained with patients in less intensive modalities (whose condition typically is less severe) could not be replicated if patients with more severe conditions were assigned to these modalities. In this case, CEA studies may underestimate the differential benefit of the more intensive treatment and for this reason may falsely conclude that less intensive care is more cost-effective. Therefore, the next generation of CEAs in alcohol service research must incorporate a deeper appreciation of the possible variability of treatment effects for various patient subgroups.

## Footnotes

1 In this article, the term *alcoholism* is used to refer to all types of excessive alcohol consumption, regardless of whether they meet the diagnostic criteria for alcohol abuse and alcohol dependence.
